# Coffee and Typhoid Fever

**Published:** 1889-09

**Authors:** 


### COFFEE AND TYPHOID FEVER.

In a practice of twenty-five years, Dr. W. A. Cusick (*Southern Med-Record*) has not observed a fatal issue in any case of typhoid fever where the patient expressed a fondness for coffee and used it freely in the course of the sickness, and hence he has encouraged its free use unless interdicted by some idiosyncrasy. He believes that coffee imparts "staying qualities" to the nervous system which, in self-limited fevers, will often tide the patient over the critical period to convalescence when he would otherwise sink and die.

